# A financial market with singular drift and no arbitrage

**DOI:** 10.1007/s11579-020-00284-9

**Published:** 2020-11-25

**Authors:** Nacira Agram, Bernt Øksendal

**Affiliations:** 1 Department of Mathematics, Linnaeus University, SE-351 95 Växjö, Sweden; 2 Department of Mathematics, University of Oslo, Box 1053 Blindern, NO-0316 Oslo, Norway

**Keywords:** Jump diffusion, Financial market with a local time drift term, Arbitrage, Optimal portfolio, Delayed information, Donsker delta function, White noise calculus, 60H05, 60H40, 93E20, 91G80, 91B70

## Abstract

We study a financial market where the risky asset is modelled by a geometric Itô-Lévy process, with a singular drift term. This can for example model a situation where the asset price is partially controlled by a company which intervenes when the price is reaching a certain lower barrier. See e.g. Jarrow and Protter (J Bank Finan 29:2803–2820, 2005) for an explanation and discussion of this model in the Brownian motion case. As already pointed out by Karatzas and Shreve (Methods of Mathematical Finance, Springer, Berlin, 1998) (in the continuous setting), this allows for arbitrages in the market. However, the situation in the case of jumps is not clear. Moreover, it is not clear what happens if there is a delay in the system. In this paper we consider a jump diffusion market model with a singular drift term modelled as the local time of a given process, and with a delay $\theta > 0$ in the information flow available for the trader. We allow the stock price dynamics to depend on both a continuous process (Brownian motion) and a jump process (Poisson random measure). We believe that jumps and delays are essential in order to get more realistic financial market models. Using white noise calculus we compute explicitly the optimal consumption rate and portfolio in this case and we show that the maximal value is finite as long as $\theta > 0$. This implies that there is no arbitrage in the market in that case. However, when $\theta$ goes to 0, the value goes to infinity. This is in agreement with the above result that is an arbitrage when there is no delay. Our model is also relevant for high frequency trading issues. This is because high frequency trading often leads to intensive trading taking place on close to infinitesimal lengths of time, which in the limit corresponds to trading on time sets of measure 0. This may in turn lead to a singular drift in the pricing dynamics. See e.g. Lachapelle et al. (Math Finan Econom 10(3):223–262, 2016) and the references therein.

## Introduction

It is well-known that in the classical Black-Scholes market, there is no arbitrage. However, if we include a singular term in the drift of the risky asset, it was first proved by Karatzas and Shreve [11] (Theorem B2, page 329), that arbitrages exist. Subsequently this type of market has been studied by several authors, including Jarrow and Protter [10]. They explain how a singular term in the drift can model a situation where the asset price is partially controlled by a large company which intervenes when the price is reaching a certain lower barrier, in order to prevent it from going below that barrier. They also prove that arbitrages can occur in such situations.

The purpose of our paper is to extend this study in two directions:

First, we introduce jumps in the market. More precisely, we study a jump diffusion market driven by a Brownian motion $B(\cdot )$ and an independent compensated random measure $\widetilde{N}(\cdot ,\cdot )$ with an added singular drift term, modelled by a local time of an underlying Lévy process $Y(\cdot )$. In view of the unstable financial markets we have seen in recent years, and in particular during the economic crises in 2008 and the corona virus crisis this year, we think that jumps are useful in an attempt to obtain more realistic financial market models.

Introducing jumps in the stock price motion goes back to Cox and Ross [5] and to Merton [12].

Second, we assume that the trader only has access to a *delayed* information flow, represented by the filtration $\mathcal {F}_{t-\theta }$, where $\theta >0$ is the delay constant and $\mathcal {F}_{t}$ is the sigma-algebra generated by both $\{B(s)\}_{s\le t}$ and $\{N(s,\cdot )\}_{s\le t}$. This extension is also motivated by the effort to get more realistic market models. Indeed, in all real-life markets there is delay in the information flow available, and traders are willing to pay to get the most recent price information. Especially, when trading with computers even fractions of seconds of delays are important. We represent the singular term by the local time of a given process and show that as long as $\theta >0$ there is no arbitrage in this market. In fact, we show that this delayed market is *viable*, in the sense that the value of the optimal portfolio problem with logarithmic utility is finite. However, if the delay goes to 0, the value of the portfolio goes to infinity, at least under some additional assumptions.

We emphasize that our paper deals with *delayed information flow*, not delay in the coefficients in the model, as for example in the paper by Arriojas *et al* [2]. There are many papers on optimal stochastic control with delayed information flow, also by us. However, to the best of our knowledge the current paper is the first to discuss the effect of delay in the information flow on arbitrage opportunities in markets with a singular drift coefficient. We will show that by applying techniques from white noise theory we can obtain explicit results. Specifically, our model is the following:

Suppose we have a financial market with the following two investment possibilities:

- A risk free investment (e.g. a bond or a (safe) bank account), whose unit price $S_{0}(t)$ at time *t* is described by
  1.1 $$\begin{aligned} {\left\{ \begin{array}{ll} dS_{0}(t)=r(t)S_{0}(t)dt;\quad t \in [0, T],\\ S_{0}(0)=1. \end{array}\right. } \end{aligned}$$
- A risky investment, whose unit price *S*(*t*) at time *t* is given by a linear stochastic differential equation (SDE) of the form
  1.2 $$\begin{aligned} {\left\{ \begin{array}{ll} dS(t) &{} \!\!=\!S(t^{-})\left[ \mu (t)dt\!+\!\alpha (t)dL_{t}\!+\!\sigma (t)dB(t)\!+\!\int _{\mathbb {R} _{0}}\gamma (t,\zeta )\widetilde{N}(dt,d\zeta )\right] ; \quad t \in [0,T],\\ S(0) &{} >0, \end{array}\right. } \end{aligned}$$

where $\mathbb {R}_0=\mathbb {R} {\setminus } \{0\}.$ Here $B(\cdot )$ and $\widetilde{N}=N(dt,d\zeta )-\nu (d\zeta )dt$ is a standard Brownian motion and an independent compensated Poisson random measure, respectively, defined on a complete filtered probability space $(\Omega ,\mathcal {F},P)$ equipped with the filtration $\mathbb {F}=\{\mathcal {F} _{t}\}_{t\ge 0}$ generated by the Brownian motion $B(\cdot )$ and $N(\cdot )$. The measure $\nu$ is the Lévy measure of the Poisson random measure *N*, and the singular term $L_{t}=L_{t}(y)$ is represented as the local time at a point $y\in \mathbb {R}$ of a given $\mathbb {F}$-predictable process $Y(\cdot )$ of the form

1.3 $$\begin{aligned} Y(t)=\int _{0}^{t}\phi (s)dB(s)+\int _{0}^{t}\int _{\mathbb {R}_{0}}\psi (s,\zeta )\tilde{N}(ds,d\zeta ), \end{aligned}$$

for some real deterministic functions $\phi : [0,T] \rightarrow \mathbb {R},\psi :[0,T]\times \mathbb {R}_0\rightarrow \mathbb {R}$ satisfying

1.4 $$\begin{aligned} 0<\int _{t}^{T}\Big \{\phi ^{2}(t)+\int _{\mathbb {R}_{0}}\psi ^{2}(t,\zeta )\nu (d\zeta )\Big \}dt<\infty \text { a.s. for all }t\in [0,T]. \end{aligned}$$

The coefficients $r(t),\mu (t),\alpha (t),$ $\sigma (t)>0$ and $\gamma (t,\zeta )>0$ are given bounded $\mathbb {F}$-predictable processes, with $\sigma (t)$ bounded away from 0.

In this market we introduce a *portfolio process* $u:[0,T]\times \Omega \rightarrow \mathbb {R}$ giving the fraction of the wealth invested in the risky asset at time *t*, and a *consumption rate process* $c:[0,T]\times \Omega \rightarrow \mathbb {R}^{+}$ giving the fraction of the wealth consumed at time *t*. We assume that at any time *t* both *u*(*t*) and *c*(*t*) are required to be adapted to a given possibly smaller filtration $\mathbb {G}=\{\mathcal {G}_{t}\}_{t\in [0,T]}$ with $\mathcal {G} _{t}\subseteq \mathcal {F}_{t}$ for all *t*. For example, it could be a delayed information flow, with

1.5 $$\begin{aligned} \mathcal {G}_{t}=\mathcal {F}_{\max (0,t-\theta )},\quad t\ge 0,\text { for some delay }\theta >0. \end{aligned}$$

This case will be discussed in detail later.

Let us denote by $\mathcal {A}_{\mathbb {G}}$ the set of all admissible consumption and portfolio processes. We say that *c* and *u* are admissible and write $c,u\in \mathcal {A}_{\mathbb {G}}$ if, in addition, *u* is self-financing and $\mathbb {E}\Big [\int _{0}^{T}(u(t)^{2}+c(t)^{2})dt\Big ]<\infty ,$ where $\mathbb {E}$ denotes expectation with respect to *P*. Note that if *c*, *u* are admissible, then the corresponding wealth process $X(t)=X^{c,u}(t)$ is described by the equation

1.6 $$\begin{aligned} dX(t)&=X(t^{-})[[(1-u(t))r(t)+u(t)\mu (t)-c(t)]dt+u(t)\alpha (t)dL_{t}\nonumber \\&\quad +u(t)\sigma (t)dB(t)+u(t)\textstyle \int _{\mathbb {R}_{0}}\gamma (t,\zeta )\widetilde{N}(dt,d\zeta )]. \end{aligned}$$

For simplicity, we put the initial value $X(0)=1.$

The optimal consumption and portfolio problem we study is the following:

### Problem 1.1

Let $a>0,b>0$ be given constants. Find admissible $c^{*},u^{*},$ such that

1.7 $$\begin{aligned} J(c^{*},u^{*})=\sup _{c,u}J(c,u), \end{aligned}$$

where

1.8 $$\begin{aligned} J(c,u)=\mathbb {E}\Big [\int _{0}^{T}a\ln (c(t)X(t))dt+b\ln (X(T))\Big ]. \end{aligned}$$

Our results are the following:

Using methods from white noise calculus we find explicit expressions for the optimal consumption rate $c^{*}(t)$ and the optimal portfolio $u^{*} (t)$. Then we show that the value is finite for all positive delays in the information flow. In particular, this shows that there is no arbitrage in that case. This result appears to be new.

We also show that, under additional assumptions, the value goes to infinity when the delay goes to 0. This shows in particular that also when there are jumps the value is infinite when there is no delay, in agreement with the arbitrage results of Karatzas and Shreve [11] and Jarrow and Protter [10] in the Brownian motion case.

### Remark 1.2

In our problem we are using the logarithmic utility function, both for the consumption and for the terminal value. It is natural to ask if similar results can be obtained for other utility functions. The method used in this paper is quite specific for the logarithmic utility and will not work for other cases. This issue will be discussed in a broader context in a future research.

## Preliminaries

As we have mentioned above, we will use white noise calculus to find explicit expressions for the optimal consumption and the optimal portfolio. Specifically, we will define the local time in the terms of the Donsker delta function which is an element of the Hida space of stochastic distributions $(\mathcal {S})^{*}$. A brief introduction to white noise calculus is given in the Appendix. For more information on the underlying white noise theory we refer to Hida et al. [9], Oliveira [14], Holden et al. [8] and Di Nunno et al. [7] and Agram and Øksendal [3].

### The Donsker delta function

We now define the Donsker delta function and give some of its properties. It will play a crucial role in our computations.

#### Definition 2.1

Let $Y:\Omega \rightarrow \mathbb {R}$ be a random variable which also belongs to the Hida space $(\mathcal {S})^{*}$ of stochastic distributions. Then a continuous functional

2.1 $$\begin{aligned} \delta _{Y}(\cdot ):\mathbb {R}\rightarrow (\mathcal {S})^{*} \end{aligned}$$

is called a *Donsker delta function* of *Y* if it has the property that

2.2 $$\begin{aligned} \int _{\mathbb {R}}g(y)\delta _{Y}(y)dy=g(Y),\quad \text {a.s.} \end{aligned}$$

for all (measurable) $g:\mathbb {R}\rightarrow \mathbb {R},$ such that the integral converges.

Explicit formulas for the Donsker delta function are known in many cases. For the Gaussian case, see Section 3.2. For details and more general cases, see e.g. Aase et al. [1].

In particular, for our process *Y* described by the diffusion (1.3), it is well known (see e.g. [6, 7, 13]) that the Donsker delta functional exists in $(\mathcal {S})^{*}$ and is given by

2.3 $$\begin{aligned} \delta _{Y(t)}(y)&=\frac{1}{2\pi }{\int _{\mathbb {R}}}\exp ^{\diamond }\left[ {\int _{0}^{t}\int _{\mathbb {R}_{0}}}(e^{ix\psi (s,\zeta )}-1)\tilde{N}(ds,d\zeta )\right. \nonumber \\&\quad +\int _{0}^{t}ix\phi (s)dB(s)\nonumber \\&\quad +\int _{0}^{t}\left\{ \int _{\mathbb {R}_{0}}(e^{ix\psi (s,\zeta )}-1-ix\psi (s,\zeta ))\nu (d\zeta )\right. \nonumber \\&\quad \left. \left. -\frac{1}{2}x^{2}\phi ^{2}(s)\right\} ds-ixy\right] dx, \end{aligned}$$

where $\exp ^{\diamond }$ denotes the Wick exponential.

Moreover, if $0\le s\le t,$ we can compute the conditional expectation

2.4 $$\begin{aligned}&\mathbb {E}[\delta _{Y(t)}(y)|\mathcal {F}_{s}]\nonumber \\&\quad =\frac{1}{2\pi }{\int _{\mathbb {R}}}\exp \big [\int _{0}^{s} \int _{\mathbb {R}_{0}}ix\psi (r,\zeta )\tilde{N}(dr,d\zeta )+\int _{0}^{s} ix\phi (r)dB(r)\nonumber \\&\qquad +{\int _{s}^{t}\int _{\mathbb {R}_{0}}}(e^{ix\psi (r,\zeta )} -1-ix\psi (r,\zeta ))\nu (d\zeta )dr-\int _{s}^{t}\frac{1}{2}x^{2}\phi ^{2}(r)dr-ixy\bigg ]dx. \end{aligned}$$

Note that if we put $s=0$ in (2.4), we get

$$\begin{aligned} \mathbb {E}[\delta _{Y(t)}(y)]&={\frac{1}{2\pi }\int _{\mathbb {R}} \exp \Big (-\frac{1}{2}x^{2}\int _{0}^{t}\phi ^{2}(r)dr}\\&\quad +{\int _{0}^{t}\int _{\mathbb {R}_{0}}(e^{ix\psi (r,\zeta )} -1-ix\psi (r,\zeta ))\nu (d\zeta )dr-ixy\Bigg )dx}<\infty . \end{aligned}$$

Putting $\nu =0$ in (2.4), yields

2.5 $$\begin{aligned}&\frac{1}{2\pi }\int _{\mathbb {R}}\exp \Big [\int _{0}^{s} ix\phi (r)dB(r)-\textstyle {\int _{s}^{t}\frac{1}{2}}x^{2}\phi ^{2} (r)dr-ixy\Big ]dx\nonumber \\&\quad =\left( 2\pi \textstyle {\int _{s}^{t}}\phi ^{2}(r)dr\right) ^{-\frac{1}{2} }\exp \Bigg (-\frac{\left( \int _{0}^{s}\phi (r)dB(r)-y\right) ^{2}}{2 \int _{s} ^{t}\phi ^{2}(r)dr}\Bigg ), \end{aligned}$$

where we have used, in general, for $a>0,b\in \mathbb {R}$, that

2.6 $$\begin{aligned} \int _{{\mathbb {R}}}e^{-ax^{2}-2bx}dx=\sqrt{\frac{\pi }{a}} e^{\frac{b^{2}}{a}}. \end{aligned}$$

In particular, applying the above to the random variable $Y(t):=B(t)$ for some $t\in (0,T]$ where *B* is Brownian motion starting at 0 , we get for all $0\le s < t$,

2.7 $$\begin{aligned} \mathbb {E}[\delta _{B(t)}(y)|\mathcal {F}_s]=(2\pi (t-s))^{-\frac{1}{2}}\exp \Big [-\frac{(B(s)-y)^{2}}{2(t-s)}\Big ]. \end{aligned}$$

We will also need the following estimate:

#### Lemma 2.2

Assume that $0\le s\le t\le T$. Then

2.8 $$\begin{aligned} \mathbb {E}[\delta _{Y(t)}(y)|\mathcal {F}_{s}]\le \Big (2\pi \textstyle {\int _{s}^{t} }\left\{ \phi ^{2}(r)+\int _{\mathbb {R}_{0}}\psi ^{2}(r,\zeta )\nu (d\zeta )\right\} dr \Big )^{-\frac{1}{2}}. \end{aligned}$$

#### Proof

From (2.4) we get, with $i=\sqrt{-1}$,

$$\begin{aligned}&|\mathbb {E}[\delta _{Y(t)}(y)|\mathcal {F}_{s}]| \le {\frac{1}{2\pi }\int _{\mathbb {R}}\exp }\left[ \int _{s} ^{t}\int _{\mathbb {R}_{0}}Re(e^{ix\psi (r,\zeta )}\right. \\&\qquad \left. -1-ix\psi (r,\zeta ))\nu (d\zeta )dr-\frac{1}{2}\int _{s}^{t}x^{2}\phi ^{2}(r)dr\right] {dx}\\&\quad \le {\frac{1}{2\pi }\int _{\mathbb {R}}\exp }\left[ {\int _{s} ^{t}\int _{\mathbb {R}_{0}}-\frac{1}{2}x^{2}\psi ^{2}(r,\zeta )\nu (d\zeta )dr-\frac{1}{2}\int _{s}^{t}x^{2}\phi ^{2}(r)dr}\right] {dx}\\&\quad =\textstyle {\frac{1}{2\pi }\int _{\mathbb {R}}\exp }\left[ {-\frac{1}{2} x^{2}\int _{s}^{t}}\left\{ {\phi ^{2}(r)+\int _{\mathbb {R}_{0}}\psi ^{2} (r,\zeta ))\nu (d\zeta )}\right\} {dr}\right] {dx}\\&\quad ={\Big (2\pi }\left( {\int _{s}^{t}}\left\{ {\phi ^{2} (r)+\int _{\mathbb {R}_{0}}\psi ^{2}(r,\zeta )\nu (d\zeta )}\right\} {dr}\right) {\Big )^{-\frac{1}{2}}}. \end{aligned}$$

$$\square $$

### Local time in terms of the Donsker delta function

In this subsection we define the local time of $Y(\cdot )$ at *y* and we give a representation of it in terms of the Donsker delta function.

#### Definition 2.3

The local time $L_{t}(y)$ of $Y(\cdot )$ at the point *y* and at time *t* is defined by

$$\begin{aligned} L_{t}(y)=\lim _{\epsilon \rightarrow 0}\frac{1}{2\epsilon }\lambda (\{s\in [0,t];Y(s)\in (y-\epsilon ,y+\epsilon )\}), \end{aligned}$$

where $\lambda$ denotes Lebesgue measure on $\mathbb {R}$ and the limit is in $L^{2}(\lambda \times P)$.

#### Remark 2.4

Note that this definition differs from the definition in Protter [15] Corollary 3, page 230, in two ways:

(i) We are using Lebesgue measure $d\lambda (s)=ds$ as integrator, not $d[Y,Y]_s$.
(ii) Protter [15] is defining left-sided and right-side local times. Our local time corresponds to the average of the two.

If the process *Y* is Brownian motion both definitions coincide with the standard one. We choose our definition because it is convenient for our purpose.

There is a close connection between local time and the Donsker delta function of *Y*(*t*), given by the following result.

#### Theorem 2.5

The local time $L_{t}(y)$ of *Y* at the point *y* and the time *t* is given by the following $\mathcal {S}^{*}$-valued integral

2.9 $$\begin{aligned} L_{t}(y)=\int _{0}^{t}\delta _{Y(s)}(y)ds, \end{aligned}$$

where the integral converges in $(\mathcal {S})^{*}$.

#### Proof

In the following we let $\chi _F$ denote the indicator function of the Borel set *F*, i.e.

2.10 $$\begin{aligned} \chi _F(x)&= {\left\{ \begin{array}{ll} 1 \text { if } x \in F,\\ 0 \text { if } x \notin F. \end{array}\right. } \end{aligned}$$

By definition of the local time and the Donsker delta function, we have

$$\begin{aligned} L_{t}(y)&=\lim _{\epsilon \rightarrow 0} \int _{0}^{t} \textstyle {\frac{1}{2\epsilon }}\chi _{(y-\epsilon ,y+\epsilon )} (Y(s))ds \\&=\lim _{\epsilon \rightarrow 0} \int _{0}^{t} \Big (\int _{\mathbb {R}}\frac{1}{2\epsilon } \chi _{(y-\epsilon ,y+\epsilon )} (x) \delta _{Y(s)}(x) dx\Big )ds\\&=\lim _{\epsilon \rightarrow 0} \int _{\mathbb {R}}\textstyle {\frac{1}{2\epsilon }} \chi _{(y-\epsilon ,y+\epsilon )} (x) \Big (\int _{0}^{t} \delta _{Y(s)}(x) ds\Big )dx =\int _{0}^{t} \delta _{Y(s)}(y) ds, \end{aligned}$$

because the function $y \mapsto \delta _{Y(s)}(y)$ is continuous in $(\mathcal {S})^{*}$. $\square$

## Optimal consumption and portfolio in a market with a local time drift term under partial information

We now return to the model in the Introduction. Thus we consider the optimal portfolio and consumption problem (1.7)–(1.8) of an agent in the financial market (1.1) and (1.2). The agent has access to a partial information flow $\mathbb {G}=\{\mathcal {G}_{t}\}_{t\ge 0}$ where $\mathcal {G}_{t}\subseteq \mathcal {F}_{t}$ for all *t*. It is known that if $\mathbb {G}=\mathbb {F}$, i.e. $\mathcal {G}_{t}=\mathcal {F}_{t}$ for all *t*, and if there are no jumps ($N=\nu =0$), then the market is complete and it allows an arbitrage. See Karatzas and Shreve [11] and Jarrow and Protter [10]. It is clear that our market with jumps is not complete, even if $\mathbb {G}=\mathbb {F}$. However, we will show that if $\mathcal {G}_{t}=\mathcal {F}_{t-\theta }$ for some delay $\theta >0$, then the market is viable (i.e. the optimal consumption and portfolio problem has a finite value) and it has no arbitrage. Moreover, we will find explicitly the optimal consumption and portfolio rates. If the delay goes to 0, we show that the value goes to infinity, in agreement with the existence of arbitrage in the no-delay case.

First we need the following auxiliary result.

### Lemma 3.1

Suppose that $\mathbb {E}[\delta _{Y(t)}(y)|\mathcal {G}_t] \in L^{2}(P)$ and that

$$\begin{aligned} \mu (t)-r(t)+\alpha (t)\mathbb {E}[\delta _{Y(t)}(y)|\mathcal {G}_{t}]>0. \end{aligned}$$

Then there exists a unique solution $u(t)=u^{*}(t)>0$ of the equation

$$\begin{aligned}&(a+b)\sigma ^{2}(t)u^{*}(t)+[a(T-t)+b]\int _{\mathbb {R}_{0} }\dfrac{u^{*}(t)\gamma ^{2}(t,\zeta )}{1+u^{*}(t)\gamma (t,\zeta )} \nu (d\zeta )\\&\quad =(a(T-t)+b)[\mu (t)-r(t)+\alpha (t)\mathbb {E}[\delta _{Y(t)}(y)|\mathcal {G}_{t}]]. \end{aligned}$$

### Proof

Define

$$\begin{aligned} F(u)=a_{1}u+a_{2}\int _{\mathbb {R}_{0}}\frac{u\gamma ^{2}(t,\zeta )}{1+u\gamma (t,\zeta )}\nu (d\zeta ),\quad u\ge 0, \end{aligned}$$

where $$a_{1}=(a+b)\sigma ^{2}(t),a_{2}=a(T-t)+b.$$ Then

$$\begin{aligned} F^{\prime }(u)=a_{1}+a_{2}\int _{\mathbb {R}_{0}}\frac{\gamma ^{2}(t,\zeta )}{(1+\gamma (t,\zeta ))^{2}}\nu (d\zeta )>0, \end{aligned}$$

and

$$\begin{aligned} F(0)=0,\quad \lim _{u\rightarrow \infty }F(u)=\infty . \end{aligned}$$

Therefore, for all $a>0$ there exists a unique $u>0$ such that $F(u)=a$. $\square$

We can now proceed to our first main result:

### Theorem 3.2

**(Optimal consumption and portfolio)** Assume that $\alpha$ and $\gamma >0$ are $\mathbb {G}$-adapted and that

$$\begin{aligned}&\mathbb {E}[\delta _{Y(t)}(y)|\mathcal {G}_{t}]\in L^{2}(\lambda \times P)\text { and } \mathbb {E}[ \mu (t)-r(t)|\mathcal {G}_t] +\alpha (t)\mathbb {E}[\delta _{Y(t)}(y)|\mathcal {G}_{t}]>0,\text { for all }t\in [0,T]. \end{aligned}$$

Then the optimal consumption rate is

$$\begin{aligned} c^{*}(t)=c^{*}(t)=\frac{a}{b+a(T-t)}, \end{aligned}$$

and the optimal portfolio is given as the unique solution $u^{*}(t)>0$ of the equation

$$\begin{aligned}&(a+b)\mathbb {E}[\sigma ^{2}(t)|\mathcal {G}_t] u^{*}(t)+(a(T-t)+b)\int _{\mathbb {R}_{0} }\frac{u^{*}(t)\gamma ^{2}(t,\zeta )}{1+u^{*}(t)\gamma (t,\zeta )}\nu (d\zeta )\\&\quad =(a(T-t)+b)\Big (\mathbb {E}[\mu (t)-r(t)|\mathcal {G}_t]+\alpha (t)\mathbb {E}[\delta _{Y(t)}(y)|\mathcal {G} _{t}]\Big ). \end{aligned}$$

In particular, if there are no jumps ($N=\nu =0$), the optimal portfolio will be

$$\begin{aligned} u^{*}(t)=\frac{(a(T-t)+b)\Big (\mathbb {E}[\mu (t)-r(t)|\mathcal {G}_t]+\alpha (t)\mathbb {E}[\delta _{Y(t)} (y)|\mathcal {G}_{t}]\Big )}{(a+b)\mathbb {E}[\sigma ^{2}(t)|\mathcal {G}_t]}. \end{aligned}$$

### Proof

By the Itô formula for semimartingales, see e.g. Protter [15], we get that the solution of (4.2) is

$$\begin{aligned} X(t)&=\exp \Big (\int _{0}^{t}u(s)\sigma (s)dB(s)\\&\quad +\int _{0}^{t}\left\{ r(s)+[\mu (s)-r(s)]u(s)-c(s)-\frac{1}{2}\sigma ^{2}(s)u^{2}(s)\right\} ds\\&\quad +\int _{0}^{t}u(s)\alpha (s)dL_{s} +\int _{0}^{t}\int _{\mathbb {R}_{0}}\{\ln (1+u(s)\gamma (s,\zeta ))-u(s)\gamma (s,\zeta )\}\nu (d\zeta )ds\\&\quad +\int _{0}^{t}\int _{\mathbb {R}_{0}}\ln \left\{ 1+u(s)\gamma (s,\zeta )\right\} \widetilde{N}(ds,d\zeta )\Big ). \end{aligned}$$

Since $\sigma$ and $\gamma$ are bounded and $u \in \mathcal {A}_{\mathbb {G}}$ the stochastic integrals in the exponent have expectation 0. Therefore we get

3.1 $$\begin{aligned} \mathbb {E}|\ln (X(t))]&=\mathbb {E}\Bigg [\int _{0}^{t}\{r(s)+[\mu (s)-r(s)]u(s)-c(s)-\frac{1}{2}\sigma ^{2}(s)u^{2}(s)\}ds\nonumber \\&\quad +\int _{0}^{t}u(s)\alpha (s)dL_{s}+\int _{0}^{t}\int _{\mathbb {R}_{0}} \{\ln (1+u(s)\gamma (s,\zeta ))-u(s)\gamma (s,\zeta )\}\nu (d\zeta )ds\Bigg ]. \end{aligned}$$

Formulas (4.2) and (1.8) and the Itô formula, lead to

$$\begin{aligned} J(c,u)&=\mathbb {E}\Bigg [\int _{0}^{T}a\ln (c(t)X(t))dt+b\ln (X(T))\Bigg ]\\&=\mathbb {E}\Big [\int _{0}^{T}\Bigg \{a\ln (c(t))+a\ln (X(t))\\&\quad +b\Bigg (r(t)+[\mu (t)-r(t)]u(t)-c(t)-\frac{1}{2}\sigma ^{2}(t)u^{2} (t)\Bigg )\Bigg \}dt\\&\quad +b\int _{0}^{T}u(t)\alpha (t)dL_{t}\\&\quad +b\int _{0}^{T}\int _{\mathbb {R}_{0}}\{\ln (1+u(t)\gamma (t,\zeta ))-u(t)\gamma (t,\zeta )\}\nu (d\zeta )dt\Bigg ]. \end{aligned}$$

Substituting (3.1) in the above, gives

$$\begin{aligned}&J(c,u)=\mathbb {E}\Big [\int _{0}^{T}\Big \{a\ln (c(t))\\&\quad +a\Big (\int _{0}^{t}\{r(s)+[\mu (s)-r(s)]u(s)-c(s)-\frac{1}{2}\sigma ^{2}(s)u^{2}(s)\}ds\\&\quad +\int _{0}^{t}u(s)\alpha (s)dL_{s}\\&\quad +\int _{0}^{t}\int _{\mathbb {R}_{0}}\{\ln (1+u(s)\gamma (s,\zeta ))-\pi (s)\gamma (s,\zeta )\}\nu (d\zeta )ds\Big )\\&\quad +b\Big (\int _{0}^{T}\Big \{r(t)+[\mu (t)-r(t)]u(t)-c(t)-\frac{1}{2}\sigma ^{2}(t)u^{2}(t)\Big )\Big \}dt\\&\quad +\int _{0}^{T}u(t)\alpha (t)dL_{t}\\&\quad +\int _{0}^{T}\int _{\mathbb {R}_{0}}\{\ln (1+u(t)\gamma (t,\zeta ))-u(t)\gamma (t,\zeta )\}\nu (d\zeta )dt\Big )\Big ]. \end{aligned}$$

Note that in general, we have, by the Fubini theorem,

$$\begin{aligned} \int _{0}^{T}\Big (\int _{0}^{t}h(s)ds\Big )dt&=\int _{0}^{T}\Big (\int _{s} ^{T}h(s)dt\Big )ds\\&=\int _{0}^{T}(T-s)h(s)ds=\int _{0}^{T}(T-t)h(t)dt, \end{aligned}$$

and

$$\begin{aligned} \int _{0}^{T}\Big (\int _{0}^{t}h(s)dL_{s}\Big )dt&=\int _{0}^{T}\Big (\int _{s}^{T}h(s)dt\Big )dL_{s}\\&=\int _{0}^{T}(T-s)h(s)dL_{s}=\int _{0}^{T}(T-t)h(t)dL_{t}. \end{aligned}$$

Therefore, using that

$$\begin{aligned} dL_{t}=dL_{t}(y)=\delta _{Y(t)}(y)dt, \end{aligned}$$

we get from the above that

3.2 $$\begin{aligned} J(c,u)&=\mathbb {E}\Big [\int _{0}^{T}E\Big [\Big \{a\Big (\ln (c(t))+(T-t)\{r(t)+[\mu (t)-r(t)]u(t) -c(t)-\frac{1}{2}\sigma ^{2}(t)u^{2}(t)\nonumber \\&\quad +(T-t)u(t)\alpha (t)\delta _{Y(t)}(y)\nonumber \\&\quad +(T-t)\int _{\mathbb {R}_{0}}\{\ln (1+u(t)\gamma (t,\zeta ))-u(t)\gamma (t,\zeta )\}\nu (d\zeta )\Big )dt\nonumber \\&\quad +b\Big (r(t)+[\mu (t)-r(t)]u(t)-c(t)-\frac{1}{2}\sigma ^{2}(t)u^{2} (t)\}+u(t)\alpha (t)\delta _{Y(t)}(y)\nonumber \\&\quad +\int _{\mathbb {R}_{0}}\{\ln (1+u(t)\gamma (t,\zeta ))-u(t)\gamma (t,\zeta )\}\nu (d\zeta )\Big )\Big \}\Big |\mathcal {G}_{t}\Big ]dt\Big ]. \end{aligned}$$

Using that $c,u,\alpha$ and $\gamma$ are $\mathbb {G}$-adapted, we obtain

3.3 $$\begin{aligned} J(c,u)&=\mathbb {E}\Bigg [\int _{0}^{T}\Big \{a\Big (\ln (c(t))+(T-t)\{\mathbb {E}[r(t)|\mathcal {G}_t]\nonumber \\&\quad +\mathbb {E}[\mu (t)-r(t)|\mathcal {G}_t]u(t)\nonumber \\&\quad -c(t)-\frac{1}{2}\mathbb {E}[\sigma ^{2}(t)|\mathcal {G}_t]u^{2}(t)\}\nonumber \\&\quad +(T-t)u(t)\alpha (t)\mathbb {E}[\delta {Y(t)}(y)|\mathcal {G}_{t}]\nonumber \\&\quad +(T-t)\int _{\mathbb {R}_{0}}\{\ln (1+u(t)\gamma (t,\zeta ))-u(t)\gamma (t,\zeta )\}\nu (d\zeta )\Bigg )\nonumber \\&\quad +b\Bigg (\mathbb {E}[r(t)|\mathcal {G}_t]+\mathbb {E}[\mu (t)-r(t)|\mathcal {G}_t]u(t)-c(t)-\frac{1}{2}\mathbb {E}[\sigma ^{2}(t)|\mathcal {G}_t]u^{2} (t)\nonumber \\&\quad +u(t)\alpha (t)\mathbb {E}[\delta _{Y(t)}(y)|\mathcal {G}_{t}]\nonumber \\&\quad +\int _{\mathbb {R}_{0}}\{\ln (1+u(t)\gamma (t,\zeta ))-u(t)\gamma (t,\zeta )\}\nu (d\zeta )\Bigg )\Bigg \}dt\Bigg ]. \end{aligned}$$

This we can maximise pointwise over all possible values $c,u\in \mathcal {A} _{\mathbb {G}}$ by maximising for each *t* the integrand. Then we get the optimal consumption rate

$$\begin{aligned} c^{*}(t)=\frac{a}{b+a(T-t)}, \end{aligned}$$

and the optimal portfolio is given as the unique solution $u^{*}(t)>0$ of the equation

$$\begin{aligned}&(a+b)\mathbb {E}[\sigma ^{2}(t)|\mathcal {G}_t]u^{*}(t)\\&\qquad +[a(T-t)+b]\textstyle \int _{\mathbb {R}_{0} }\frac{u^{*}(t)\gamma ^{2}(t,\zeta )}{1+u^{*}(t)\gamma (t,\zeta )}\nu (d\zeta )\\&\quad =(a(T-t)+b)\Big [\mathbb {E}[\mu (t)-r(t)|\mathcal {G}_t]\\&\qquad +\alpha (t)\mathbb {E}[\delta _{Y(t)}(y)|\mathcal {G} _{t}]\Big ]. \end{aligned}$$

In particular, if there are no jumps ($N=\nu =0$), we get

$$\begin{aligned} u^{*}(t)=\frac{(a(T-t)+b)\Big [\mathbb {E}[\mu (t)-r(t)|\mathcal {G}_t]+\alpha (t)\mathbb {E}[\delta _{Y(t)} (y)|\mathcal {G}_{t}]\Big ]}{(a+b)\mathbb {E}[\sigma ^{2}(t)|\mathcal {G}_t]}. \end{aligned}$$

$$\square $$

###  The case when $\mathcal {G}_{t}=\mathcal {F}_{t-\theta }, \quad t \ge 0$

From now on we restrict ourselves to the subfiltration $\mathcal {G} _{t}=\mathcal {F}_{t-\theta },t\ge 0$ for some constant delay $\theta >0$, where we put $\mathcal {F}_{t-\theta }=\mathcal {F}_{0}$ for $t\le \theta$. In this case we can compute the optimal portfolio and the optimal consumption explicitly. By (2.4) we have the following result:

#### Lemma 3.3

Assume that $\alpha$ and $\gamma > 0$ are $\mathbb {G}_{\theta }$-adapted, where $\mathbb {G}_{\theta } =\{ \mathcal {F}_{t-\theta }\}_{t\ge 0}$. For $t \ge \theta$ we have

3.4 $$\begin{aligned} \mathbb {E}\left[ \delta _{Y(t)}(y)|\mathcal {F}_{t-\theta }\right]&={\frac{1}{2\pi } \int _{\mathbb {R}}}\nonumber \\&\quad \exp \left[ \int _{0}^{t-\theta }\int _{\mathbb {R}_{0}} ix\psi (r,\zeta )\widetilde{N}(dr,d\zeta )+\int _{0}^{t-\theta }ix\phi (r)dB(r)\right. \nonumber \\&\quad +{\int _{t-\theta }^{t}}\int _{\mathbb {R}_{0}}(e^{ix\psi (r,\zeta )}-1-ix\psi (r,\zeta ))\nu (d\zeta )dr\nonumber \\&\left. \quad -\int _{t-\theta }^{t}\frac{1}{2}x^{2}\phi ^{2}(r)dr-ixy\right] dx. \end{aligned}$$

In particular, if $\psi =0$ and $\phi =1$, we get $Y=B$ and (see also (2.7))

3.5 $$\begin{aligned} \mathbb {E}[\delta _{B(t)}(y)|\mathcal {F}_{t-\theta }]=(2\pi \theta )^{-\frac{1}{2}} \exp \Big [-\frac{(B(t-\theta )-y)^{2}}{2\theta }\Big ]. \end{aligned}$$

Then by Theorem 3.2, we get

#### Theorem 3.4

Suppose $\mathcal {G}_{t}=\mathcal {F}_{t-\theta }$ with $\theta >0.$ Then the optimal consumption rate is given by

$$\begin{aligned} c^{*}(t)=\frac{a}{b+a(T-t)}, \end{aligned}$$

and the optimal portfolio is given as the unique solution $u^{*}(t)>0$ of the equation

$$\begin{aligned}&(a+b)\mathbb {E}[\sigma ^{2}(t)|\mathcal {F}_{t-\theta }]u^{*}(t)+(a(T-t)+b)\int _{\mathbb {R}_{0} }\frac{u^{*}(t)\gamma ^{2}(t,\zeta )}{1+u^{*}(t)\gamma (t,\zeta )}\nu (d\zeta )\\&\quad =(a(T-t)+b)\big (\mathbb {E}[\mu (t)-r(t)|\mathcal {F}_{t-\theta }]+\alpha (t)\mathbb {E}[\delta _{Y(t)}(y)|\mathcal {F} _{t-\theta }]\big ). \end{aligned}$$

In particular,

$$\begin{aligned} \sup _{c,u}J(c,u)=J(c^{*},u^{*})<\infty , \end{aligned}$$

and there is no arbitrage in the market.

## The limiting case when the delay goes to 0

In this section, we concentrate on the delay case and with optimal portfolio only, i.e. without consumption. Thus we are only considering utility from terminal wealth, and we put $a=0$ and $b=1$ in Theorem 3.2. Moreover, we assume that $\phi =1$ and $\psi =0$, i.e. that

4.1 $$\begin{aligned} Y(t)=B(t); \quad t \in [0,T]. \end{aligned}$$

Also, to simplify the calculations we assume that $r=0$ and $\mu (t)=\mu>0,\alpha (t)=\alpha>0,\sigma (t)=\sigma >0$ are constants, and $\gamma (t,\zeta )=\gamma (\zeta )$ is deterministic and does not depend on *t*. Then the wealth equation will take the form

4.2 $$\begin{aligned} dX(t)&=X(t)[u(t)\mu dt+u(t)\alpha dL_{t}\nonumber \\&\quad +u(t)\sigma dB(t)+u(t)\textstyle \int _{\mathbb {R}_{0}}\gamma (\zeta )\widetilde{N}(dt,d\zeta )];\quad t \in [0,T],\quad X(0)=1, \end{aligned}$$

where the singular term $L_{t}=L_{t}(y)$ is represented as the local time at a point $y\in \mathbb {R}$ of $B(\cdot )$. The performance functional becomes

$$\begin{aligned} J_{0}(u)=\mathbb {E}[\ln X^{(u)}(T)];\quad u\in \mathcal {A}_{\theta }, \end{aligned}$$

where $\mathcal {A}_{\theta }$ denotes the set of all $\mathcal {F}_{t-\theta }$ -predictable control processes. This now gets the form

4.3 $$\begin{aligned} J_{0}(u)&=\mathbb {E}\Big [\int _{0}^{T}\Big \{\mu u(t)-\frac{1}{2}\sigma ^{2} u^{2}(t)+u(t)\alpha \mathbb {E}[\delta _{Y(t)}(y)|\mathcal {F}_{t-\theta }]\nonumber \\&\quad +\int _{\mathbb {R}_{0}}\{\ln (1+u(t)\gamma (\zeta ))-u(t)\gamma (\zeta )\}\nu (d\zeta )\Big \}dt\Big ]. \end{aligned}$$

Our second main result is the following:

### Theorem 4.1

Suppose in addition to the above that

$$\begin{aligned} \int _{\mathbb {R}_{0}}\gamma ^{2}(\zeta )\nu (d\zeta )<\sigma ^{2}. \end{aligned}$$

Then

$$\begin{aligned} \lim _{\theta \rightarrow 0^{+}}\sup _{u\in \mathcal {A}_{\theta }}J_{0}(u)=\infty . \end{aligned}$$

In particular, if there is no delay ($\theta =0$) the value of the optimal portfolio problem is infinite.

### Proof

For given $\theta >0$ choose

$$\begin{aligned} u_{\theta }(t)=\frac{\mu +\alpha R}{\sigma ^{2}}, \end{aligned}$$

where we for simplicity put

$$\begin{aligned} R=R_{\theta }=\mathbb {E}[\delta _{B(t)}(y)|\mathcal {F}_{t-\theta }]. \end{aligned}$$

Then we see that

$$\begin{aligned} J_{0}(u_{\theta })\ge \frac{1}{2}\mathbb {E}[(\mu +\alpha R)^{2}]\Big (1-{\frac{\int _{\mathbb {R}_{0}}\gamma ^{2}(\zeta )\nu (d\zeta )}{\sigma ^{2}}}\Big )=:C_{1} \mathbb {E}[(\mu +\alpha R)^{2}]\ge C_{2}+C_{3}\mathbb {E}[R^{2}], \end{aligned}$$

since, by (2.8), $\mathbb {E}[R]<\infty$. Here $C_{1},C_{2},C_{3}$ are positive constants.

It remains to prove that

$$\begin{aligned} \mathbb {E}[R_{\theta }^{2}]\rightarrow \infty \text { when }\theta \rightarrow 0^{+}. \end{aligned}$$

To this end, note that by (2.5) we have

4.4 $$\begin{aligned} \mathbb {E}[R_{\theta }^{2}]=\mathbb {E}[(\delta _{B(t)}(y)|\mathcal {F}_{t-\theta })^{2}]=(2\pi \theta )^{-1}\mathbb {E}\Big [\exp \Big (-\frac{2(B(t-\theta )-y)^{2}}{2\theta }\Big )\Big ]. \end{aligned}$$

By formula 1.9.3(1) p.168 in [4] we have, with $\kappa >0$ constant,

4.5 $$\begin{aligned} \mathbb {E}[\exp (-\kappa (B(t-\theta )-y)^{2})]=\frac{1}{1+2\kappa (t-\theta )} \exp \Big (-\frac{\kappa y^{2}(t-\theta )}{1+2\kappa (t-\theta )}\Big ). \end{aligned}$$

Applying this to $\kappa =\frac{1}{\theta }$ we get

4.6 $$\begin{aligned} \mathbb {E}[R_{\theta } ^2]= \frac{1}{2\pi \sqrt{\theta } \sqrt{2t-\theta } } \exp \Big ( -\frac{y^2}{2t-\theta } \Big )\rightarrow \infty , \end{aligned}$$

when $$\theta \rightarrow 0$$. $$\square $$

## The Brownian motion case

In the case when $Y(t)=B(t)$ the computations above can be made more explicit. We now illustrate this, assuming for simplicity that $y=0$. Then by Theorem 3.4 the optimal portfolio $\widehat{u}(t)$ is given by

$$\begin{aligned} \widehat{u}(t)=\frac{\mu +\alpha \Lambda (t)}{\sigma ^{2}}, \end{aligned}$$

where

5.1 $$\begin{aligned} \Lambda (t)&=\mathbb {E}[\delta _{B(t)}(0)|\mathcal {F}_{t-\theta }]=(2\pi \theta )^{-\frac{1}{2}}\exp \Big [-\frac{B(t-\theta )^{2}}{2\theta }\Big ];\quad t\ge \theta ,\nonumber \\ \Lambda (t)&=\frac{1}{\sqrt{2\pi \theta }};\quad 0\le t\le \theta . \end{aligned}$$

By (4.3) and (5.1) we see, after some algebraic operations, that the corresponding performance $\widehat{J}_{\theta }=J(0,\widehat{\pi })$ is

$$\begin{aligned} \widehat{J}_{\theta }&=\mathbb {E}\Big [\int _{0}^{T}\Big (\mu \widehat{\pi }(t)-\frac{1}{2}\sigma ^{2}\widehat{\pi }^{2}(t)+\widehat{\pi }(t)\alpha \Lambda (t)\Big )dt\Big ]=\mathbb {E}\Big [\int _{0}^{T}\frac{(\mu +\alpha \Lambda (t))^{2}}{2\sigma ^{2}}dt\Big ]\\&=A_{1}+A_{2}+A_{3}, \end{aligned}$$

where

$$\begin{aligned} A_{1}=\frac{\mu ^{2}}{2\sigma ^{2}}T,\quad A_{2}=\frac{\mu \alpha }{\sigma ^{2} }\mathbb {E}\Big [\int _{0}^{T}\Lambda (t)dt\Big ],\quad A_{3}=\frac{\alpha ^{2}}{2\sigma ^{2}}\mathbb {E}\Big [\int _{0}^{T}\Lambda ^{2}(t)dt\Big ]. \end{aligned}$$

Using the density of *B*(*s*), we get

5.2 $$\begin{aligned} \mathbb {E}\Big [\exp \Big (-\frac{B^{2}(s)}{2\theta }\Big )\Big ]&=\int _{\mathbb {R}} \exp \left( -\frac{y^{2}}{2\theta }\right) \frac{1}{\sqrt{2\pi s}}\exp \left( -\frac{y^{2}}{2s}\right) dy\nonumber \\&=\frac{1}{\sqrt{2\pi s}}\int _{\mathbb {R}}\exp \left( -\frac{1}{2} y^{2}(\frac{1}{\theta }+\frac{1}{s})\right) dy. \end{aligned}$$

In general we have, for $a>0$,

$$\begin{aligned} \int _{\mathbb {R}}\exp (-ay^{2})dy=\sqrt{\frac{\pi }{a}}, \end{aligned}$$

we conclude, by putting $s=t-\theta$ in (5.2), that

$$\begin{aligned} A_{2}=\frac{\theta }{\sqrt{2\pi \theta }}+\int _{\theta }^{T}\frac{\mu \alpha }{\sigma ^{2}\sqrt{2\pi \theta }}\sqrt{\frac{\theta }{t}}dt=\sqrt{\frac{\theta }{2\pi }}+\frac{2\mu \alpha (\sqrt{T}-\sqrt{\theta })}{\sigma ^{2}\sqrt{2\pi }}. \end{aligned}$$

Finally we use similar calculations to compute

$$\begin{aligned} A_{3}=\frac{\alpha ^{2}}{2\sigma ^{2}}(2\pi \theta )^{-1}\left( \theta +\int _{\theta }^{T}\Psi (t)dt\right) , \end{aligned}$$

where, putting $$t-\theta =s$$,

$$\begin{aligned} \psi (t)&=\mathbb {E}\left[ \exp \left( -\frac{B(s)^{2}}{\theta }\right) \right] =\int _{\mathbb {R}}e^{-\frac{y^{2}}{\theta }}\frac{1}{\sqrt{2\pi s}} e^{-\frac{y^{2}}{2s}}dy\\&=\frac{1}{\sqrt{2\pi s}}\int _{\mathbb {R}}\exp \left( -y^{2}(\frac{1}{\theta }+\frac{1}{2s})\right) dy=\frac{1}{\sqrt{2\pi s}}\sqrt{\frac{\pi }{\frac{1}{\theta }+\frac{1}{2s}}}=\frac{1}{\sqrt{\frac{2s}{\theta }+1}}. \end{aligned}$$

This gives

$$\begin{aligned} A_{3}=\frac{\alpha ^{2}}{2\sigma ^{2}}\left( \frac{1}{2\pi }+\int _{0}^{T-\theta }\frac{1}{2\pi \sqrt{\theta }\sqrt{2s+\theta }}ds\right) =\frac{\alpha ^{2}}{4\pi \sigma ^{2}}\left( 1+\frac{\sqrt{2T-\theta }-\sqrt{\theta }}{\sqrt{\theta } }\right) . \end{aligned}$$

We have proved the following:

### Theorem 5.1

The optimal performance with a given delay $\theta > 0$ is given by

$$\widehat{J}_{\theta }=\frac{\mu ^{2}}{2\sigma ^{2}}T+\sqrt{\frac{\theta }{2\pi } }+\frac{2\mu \alpha (\sqrt{T}-\sqrt{\theta })}{\sigma ^{2}\sqrt{2\pi }} +\frac{\alpha ^{2}}{4\pi \sigma ^{2}}(1+\frac{\sqrt{2T-\theta }-\sqrt{\theta } }{\sqrt{\theta }}).$$

In particular, $\widehat{J}_{\theta }\rightarrow \infty$ when $\theta \rightarrow 0.$

### Corollary 5.2

(i) For all information delays $\theta >0$ the value of the optimal portfolio problem is finite.
(ii) When there is no information delay, i.e. when $\theta =0$, the value is infinite.

## Appendix

In this section we give a brief survey of the underlying theory of white noise analysis used in this paper. For more details see e.g. Di Nunno et al. [7] and Holden et al. [8] and the references therein.

### Definition 6.1

Let $\mathcal {S}(\mathbb {R})$ be the Schwartz space consisting of all real-valued rapidly decreasing functions *f* on $\mathbb {R},$ i.e.,

6.1 $$\begin{aligned} \lim _{|x|\rightarrow \infty }|x^{n}f^{(k)}(x)|=0;\quad \text {for all integers } n,k . \end{aligned}$$

### Example 6.2

For instance $\mathcal {C}^{\infty }$ functions with compact support, $f(x)=e^{-x^{2}},f(x)=e^{-x^{4}},\ldots$ are all functions in $\mathcal {S} (\mathbb {R})$.

For any $n,k\ge 0,$ define a norm $\Vert .\Vert _{n,k}$ on $\mathcal {S} (\mathbb {R})$ by

6.2 $$\begin{aligned} \Vert f\Vert _{n,k}=\sup _{x\in \mathbb {R}}(1+|x|)^{n}|f^{(k)}(x)|. \end{aligned}$$

Then the Schwartz space $\mathcal {S}(\mathbb {R)}$, equipped with the topology defined by the family of seminorms

$\{\Vert .\Vert _{n,k},n,k\ge 0\}$ is a Fréchet space.

Let $\mathcal {S}^{\prime }(\mathbb {R})$ be the dual space of $\mathcal {S} (\mathbb {R})$. $\mathcal {S}^{\prime }(\mathbb {R})$ is called the space of tempered distributions. Let $\mathcal {B}$ denote the family of all Borel subsets of $\mathcal {S}(\mathbb {R})$ equipped with the weak topology.

From now on we will use the notation $\langle a,b\rangle$ that means *a* acting on *b*.

### Theorem 6.3

**(Minlos)** Let $\mathbf {E}$ be a Fréchet space with dual space $\mathbf {E} ^{*}$. A complex-valued function $\phi$ on $\mathbf {E}$ is the characteristic functional of a probability measure $\nu$ on $\mathbf {E}^{*}$, i.e.,

6.3 $$\begin{aligned} \phi (y)=\int _{\mathbf {E}^{*}}e^{i\langle x,y\rangle }d\nu (x);\quad y\in \mathbf {E,} \end{aligned}$$

if and only if it satisfies the following conditions:

1. $$\phi (0)=1$$,
2. $\phi$ is positive definite, i.e.
  $$\begin{aligned} \sum _{j,k=1}^{n}z_{j}\bar{z}_{k}\phi (a_{j}-a_{k})\ge 0\text { for all } z_{j},z_{k}\in \mathbb {C},a_{j},a_{k}\in \mathbf {E}, \end{aligned}$$
3. $$\phi $$ is continuous.

### Remark 6.4

The measure $\nu$ is uniquely determined by $\phi$. Observe that $\phi (0)=\nu (\mathbf {E}^{*}).$ Thus when condition 1 above is not assumed, then we can only conclude that $\nu$ is a finite measure.

### White noise for Brownian motion

#### Construction of Brownian motion

Let $\phi$ be the function on $\mathcal {S}(\mathbb {R})$ given by

$$\begin{aligned} \phi (\xi )=\exp (-\frac{1}{2}|\xi |^{2});\quad \xi \in \mathcal {S}(\mathbb {R}), \end{aligned}$$

where $|\cdot |$ is the $L^{2}(\mathbb {R})$ norm.

Then it is easy to check that conditions 1–3 above are satisfied.

Therefore, by the Minlos theorem there exists a unique probability measure *P* on $\mathcal {S} ^{\prime }(\mathbb {R})$ such that

6.4 $$\begin{aligned} \exp \left( -\frac{1}{2}|\xi |^{2}\right) =\int _{\mathcal {S}^{\prime }(\mathbb {R})}e^{i\langle \omega ,\xi \rangle }dP(\omega );\quad \xi \in \mathcal {S} (\mathbb {R}). \end{aligned}$$

##### Definition 6.5

The measure *P* is called the standard Gaussian measure on $\mathcal {S} ^{\prime }(\mathbb {R})$. The probability space $(\mathcal {S}^{\prime }(\mathbb {R}),\mathcal {B},P)$ is called the white noise probability space. In the following we will use the notation $\Omega =\mathcal {S}^{\prime } (\mathbb {R})$ and the elements of $\Omega$ are denoted by $\omega$. The expectation with respect to *P* is denoted by $\mathbb {E}[\cdot ]$,

Note that from (6.4) it follows that

6.5 $$\begin{aligned} \mathbb {E}[\left\langle \omega ,\xi \right\rangle ]&=0\text { for all }\xi \in \mathcal {S}(\mathbb {R})\text { and } \end{aligned}$$

6.6

Using the Itô isometry we see that we can extend the definition of $\left\langle \omega ,\xi \right\rangle$ from $\xi \in \mathcal {S}(\mathbb {R})$ to all $\phi \in L^{2}(\mathbb {R})$ as follows:

$$\begin{aligned} \left\langle \omega ,\phi \right\rangle =\lim _{n\rightarrow \infty }\left\langle \omega ,\xi _{n}\right\rangle \text { (limit in }L^{2}(P)), \end{aligned}$$

for any sequence $\xi _{n}\in \mathcal {S}(\mathbb {R})$ converging to $\phi$ in $L^{2}(\mathbb {R}).$

Thus for each *t* we can define $B(t,\cdot )\in L^{2}(P)$ by

$$\begin{aligned} B(t,\omega )=\left\langle \omega ,\chi _{[0,t]}(\cdot \right\rangle );\quad t\ge 0,\omega \in \Omega . \end{aligned}$$

Then the process $\{B(t,\omega )\}_{t\ge 0,\omega \in \Omega }$ has stationary independent increments of mean 0 (by (6.5)), and the variance of *B*(*t*) is *t* (by (6.6)). Moreover, by the Kolmogorov continuity theorem the process has a continuous version. This version is a Brownian motion. This is the Brownian motion we work with in this paper.

#### The Wiener-Itô chaos expansion

Let the Hermite polynomials $h_{n}(x)$ be defined by

$$\begin{aligned} h_{n}(x)=(-1)^{n}e^{\frac{1}{2}x^{2}}\frac{d^{n}}{dx^{n}}(e^{-\frac{1}{2} x^{2}});\quad n=0,1,2,\ldots \end{aligned}$$

The first Hermite polynomials are

$$\begin{aligned} h_{0}(x)=1,\quad h_{1}(x)=x,\quad h_{2}(x)=x^{2}-1,\quad h_{3}(x)=x^{3} -x,\ldots \end{aligned}$$

Let $e_{k}$ be the *k*th Hermite function defined by

6.7 $$\begin{aligned} e_{k}(x):=\pi ^{-\frac{1}{4}}((k-1)!)^{-\frac{1}{2}}e^{-\frac{1}{2}x^{2} }h_{k-1}(\sqrt{2}x);\quad k=1,2,\ldots \end{aligned}$$

Then $\{e_{k}\}_{k\ge 1}$ constitutes an orthonormal basis for $L^{2}(R)$ and $e_{k}\in \mathcal {S}(\mathbb {R})$ for all *k*. Define

6.8 $$\begin{aligned} \theta _{k}(\omega ):=\langle \omega ,e_{k}\rangle =\int _{\mathbb {R}} e_{k}(x)dB(x,\omega );\quad \omega \in \Omega . \end{aligned}$$

Let $\mathcal {J}$ denote the set of all finite multi-indices $\alpha =(\alpha _{1},\alpha _{2},\ldots ,\alpha _{m}),m=1,2,\ldots ,$ of non-negative integers $\alpha _{i}.$ If $\alpha =(\alpha _{1},\ldots ,\alpha _{m})\in \mathcal {J},\alpha \ne 0,$ we put

6.9 $$\begin{aligned} H_{\alpha }(\omega ):=\prod _{j=1}^{m}h_{\alpha _{j}}(\theta _{j}(\omega ));\quad \omega \in \Omega . \end{aligned}$$

By a result of Itô we have that

6.10 $$\begin{aligned} I_{m}(e^{\widehat{\otimes }\alpha })=\prod _{j=1}h_{\alpha _{j}}(\theta _{j})=H_{\alpha }, \end{aligned}$$

where $I_{m}$ denotes the m-iterated Itô integral, defined below.

We set $H_{0}:=1$. Here and in the sequel the functions $e_{1},e_{2},\ldots$ are defined in (6.7) and $\otimes$ and $\widehat{\otimes }$ denote the tensor product and the symmetrized tensor product, respectively.

The family $\{H_{\alpha }\}_{\alpha \in \mathcal {J}}$ is an orthogonal basis for the Hilbert space $L^{2}(P)$. In fact, we have the following result.

##### Theorem 6.6

**(The Wiener-Itô chaos expansion theorem (I))** The family $\{H_{\alpha }\}\alpha \in \mathcal {J}$ constitutes an orthogonal basis of $L^{2}(P)$. More precisely, for all $\mathcal {F}_{T}$-measurable $X\in L^{2}(P)$ there exist (uniquely determined) numbers $c_{\alpha }\in \mathbb {R},$ such that

6.11 $$\begin{aligned} X=\sum _{\alpha \in \mathcal {J}}c_{\alpha }H_{\alpha }\in L^{2} (P). \end{aligned}$$

Moreover, we have

6.12 $$\begin{aligned} \Vert X\Vert _{L^{2}(P)}^{2}=\sum _{\alpha \in \mathcal {J}}\alpha !c_{\alpha } ^{2}. \end{aligned}$$

Let us compare the above Theorem to the equivalent formulation of this theorem in terms of iterated Itô integrals. In fact, if $\psi (t_{1},t_{2},\ldots ,t_{n})$ is a real deterministic symmetric function in its *n* variables $t_{1} ,\ldots ,t_{n}$ and $\psi \in L^{2}(\mathbb {R}^{n}),$ that is,

$$\begin{aligned} \Vert \psi \Vert _{L^{2}(\mathbb {R}^{n})}:=\int _{\mathbb {R}^{n}}|\psi (t_{1},t_{2},\ldots ,t_{n})|^{2}dt_{1}dt_{2}\ldots dt_{n} \end{aligned}$$

then its *n*-iterated Itô integral is defined by

$$\begin{aligned} I_{n}(\psi )&:=\int _{\mathbb {R}^{n}}\psi dB^{\otimes n}\\&=n!\int _{-\infty }^{\infty }\int _{-\infty }^{t_{n}}\int _{-\infty }^{t_{n-1} }\ldots \int _{-\infty }^{t_{2}}\psi (t_{1},t_{2},\ldots ,t_{n})dB(t_{1})dB(t_{2} )\ldots dB(t_{n}), \end{aligned}$$

where the integral on the right-hand side consists of *n*-iterated Itô integrals.

Note that the integrand at each step is adapted to the filtration $\mathbb {F}$. Applying the Itô isometry *n* times we see that

6.13 $$\begin{aligned} \mathbb {E}\Big [\left( \int _{\mathbb {R}^{n}}\psi dB^{\otimes n}\right) ^{2} \Big ]=n!\Vert \psi \Vert _{L^{2}(\mathbb {R}^{n})}^{2}. \end{aligned}$$

For $n=0$ we adopt the convention that

$$\begin{aligned} I_{0}(\psi ):=\int _{\mathbb {R}^{0}}\psi dB^{\otimes 0}=\psi =\Vert \psi \Vert _{L^{2}(\mathbb {R}^{0})}, \end{aligned}$$

for $\psi$ constant. Let $\widetilde{L}^{2}(\mathbb {R}^{n})$ denote the set of symmetric real functions on $\mathbb {R}^{n}$, which are square integrable with respect to Lebesgue measure.

Then we have the following result:

##### Theorem 6.7

[The Wiener Itô chaos expansion theorem (II)]For all $\mathcal {F}_{T}$- measurable $X\in L^{2}(P)$ there exist (uniquely determined) deterministic functions $f_{n}\in \widetilde{L}^{2}(\mathbb {R} ^{n})$ such that

6.14 $$\begin{aligned} X=\sum _{n=0}^{\infty }\int _{\mathbb {R}^{n}}f_{n}dB^{\otimes n}=\sum _{n=0}^{\infty }I_{n}(f_{n})\in L^{2}(P). \end{aligned}$$

Moreover, we have the isometry

6.15 $$\begin{aligned} \Vert X\Vert _{L^{2}(P)}^{2}=\sum _{n=0}^{\infty }n!\Vert f_{n}\Vert _{L^{2}(\mathbb {R}^{n})}^{2}. \end{aligned}$$

The connection between these two expansions in Theorems (6.6) and (6.7) is given by

$$\begin{aligned} f_{n}=\sum _{\alpha \in \mathcal {J},|\alpha |=n}c_{\alpha }e_{1}^{\otimes \alpha _{1}}\widehat{\otimes }e_{2}^{\otimes \alpha _{2}}\widehat{\otimes } \ldots \widehat{\otimes }e_{m}^{\otimes \alpha _{m}},\quad n=0,1,2,\ldots \end{aligned}$$

where $$|\alpha |=\alpha _{1}+\alpha _{2}\ldots +\alpha _{m}$$ for $$\alpha =(\alpha _{1},\ldots ,\alpha _{m})\in \mathcal {J},m=1,2,\ldots $$

Recall that the functions $e_{1},e_{2},\ldots$ are defined in (6.7) and $\otimes$ and $\widehat{\otimes }$ denote the tensor product and the symmetrized tensor product, respectively.

Note that since $H_{\alpha }=I_{m}(e^{\widehat{\otimes }\alpha }),$ for $\alpha \in \mathcal {J},|\alpha |=m,$ we get that

6.16 $$\begin{aligned} m!\Vert e^{\widehat{\otimes }\alpha }\Vert _{L^{2}(\mathbb {R}^{m})}^{2} =\alpha !, \end{aligned}$$

by combining (6.12) and (6.15) for $X=X_{\alpha }$.

#### Stochastic distribution spaces

Analogous to the test functions $\mathcal {S}(\mathbb {R})$ and the tempered distributions $\mathcal {S}^{\prime }(\mathbb {R})$ on the real line $\mathbb {R},$ there is a useful space of stochastic test functions $(\mathcal {S})$ and a space of stochastic distributions $(\mathcal {S})^{*}$ on the white noise probability space. We now explain this in detail:

In the following we use the notation

6.17 $$\begin{aligned} (2\mathbb {N})^{\alpha }=\prod _{j=1}^{m}(2j)^{\alpha _{j}}, \end{aligned}$$

if $$\alpha =(\alpha _{1},\alpha _{2},\ldots )$$.

We define

$$\begin{aligned} \varepsilon ^{(k)}=(0,0,\ldots ,1,\ldots ), \end{aligned}$$

with 1 on the *k*th place. Thus we see that

$$\begin{aligned} (2\mathbb {N})^{\varepsilon ^{(k)}}=2k. \end{aligned}$$

**The Kondratiev Spaces**  $(\mathcal {S})_{1}, (\mathcal {S})_{-1}$  **and the Hida Spaces** $(\mathcal {S})$ **and** $(\mathcal {S})^{*}$

##### Definition 6.8

Let $\rho$ be a constant in [0, 1].

- Let $k\in \mathbb {R}$. We say that $f=\sum _{\alpha \in \mathcal {J} }a_{\alpha }H_{\alpha }\in L^{2}(P)$ belongs to the Kondratiev test function Hilbert space $(\mathcal {S})_{k,\rho }$ if
  6.18 $$\begin{aligned} \Vert f\Vert _{k,\rho }^{2}:=\sum _{\alpha \in \mathcal {J}}a_{\alpha }^{2} (\alpha !)^{1+\rho }(2\mathbb {N})^{\alpha k}<\infty . \end{aligned}$$
- We define the Kondratiev test function space $(\mathcal {S})_{\rho }$ as the space
  $$\begin{aligned} (\mathcal {S})_{\rho }= \bigcap _{k\in \mathbb {R}}(\mathcal {S})_{k,\rho } \end{aligned}$$
  equipped with the projective topology, that is, $f_{n}\rightarrow f, n\rightarrow \infty ,$ in $(\mathcal {S})_{\rho }$ if and only if $\Vert f_{n} -f\Vert _{k,\rho }\rightarrow 0,$ $n\rightarrow \infty ,$ for all *k*.
- Let $q\in \mathbb {R}$. We say that the formal sum $F = \sum _{\alpha \in \mathcal {J}}b_{\alpha }H_{\alpha }$ belongs to the Kondratiev stochastic distribution space $(\mathcal {S})_{-q,-\rho }$ if
  6.19 $$\begin{aligned} \Vert f\Vert ^{2}_{-q,-\rho }:=\sum _{\alpha \in \mathcal {J} }b_{\alpha }^{2}(\alpha !)^{1-\rho }(2\mathbb {N})^{-\alpha q}<\infty . \end{aligned}$$
  We define the Kondratiev distribution space $(\mathcal {S})_{-\rho }$ by
  $$\begin{aligned} (\mathcal {S})_{-\rho } = \bigcup _{q\in \mathbb {R}}(\mathcal {S})_{-q,-\rho } \end{aligned}$$
  equipped with the inductive topology, that is, $F_{n}\rightarrow F, n\rightarrow \infty ,$ in $(\mathcal {S})_{-\rho }$ if and only if there exists *q* such that $\Vert F_{n}-F\Vert _{-q,-\rho }\rightarrow 0, n\rightarrow \infty .$
- If $\rho =0$ we write
  $$\begin{aligned} (\mathcal {S})_{0}=(\mathcal {S})\text { and }(\mathcal {S})_{-0}=(\mathcal {S} )^{*}. \end{aligned}$$
  These spaces are called the *Hida test function space* and *the Hida distribution space*, respectively.

- If $F=\sum _{\alpha \in \mathcal {J}}b_{\alpha }H_{\alpha }$ in $(\mathcal {S} )_{-1}$, we define the generalized expectation $\mathbb {E}[F]$ of *F* by
  6.20 $$\begin{aligned} \mathbb {E}[F]=b_{0}. \end{aligned}$$
  (Note that if $F\in L^{2}(P)$, then the generalized expectation coincides with the usual expectation, since $\mathbb {E}[H_{\alpha }]=0$ for all $\alpha \ne 0$).

Note that $(\mathcal {S})_{-1}$ is the dual of $(\mathcal {S})_{1}$ and $(\mathcal {S})^{*}$ is the dual of $(\mathcal {S})$. The action of $F=\sum _{\alpha \in \mathcal {J}}b_{\alpha }H_{\alpha }\in (\mathcal {S})_{-1}$ on $f=\sum _{\alpha \in \mathcal {J}}a_{\alpha }H_{\alpha }\in (\mathcal {S})_{1}$ is given by

$$\begin{aligned} \langle F,f\rangle =\sum _{\alpha }\alpha !a_{\alpha }b_{\alpha }. \end{aligned}$$

We have the inclusion

$$\begin{aligned} (\mathcal {S})_{1}\subset (\mathcal {S})\subset L^{2}(P)\subset (\mathcal {S} )^{*}\subset (\mathcal {S})_{-1}. \end{aligned}$$

##### Example 6.9

Since

$$\begin{aligned} B(t)&=\left\langle \omega ,\chi _{[0,t]}\right\rangle =\sum _{k=1}^{\infty }(e_{k},\chi _{[0,t]})\left\langle \omega ,e_{k} \right\rangle \\&=\sum _{k=1}^{\infty }\left( \int _{0}^{t}e_{k}(s)ds\right) H_{\varepsilon ^{(k)}}, \end{aligned}$$

we see that *white noise* $\overset{\bullet }{B}(t)$ defined by

$$\begin{aligned} \overset{\bullet }{B}(t)=\frac{d}{dt}B(t)=\sum _{k=1}^{\infty }e_{k} (t)H_{\varepsilon ^{(k)}}, \end{aligned}$$

exists in $$(\mathcal {S})^{*}$$.

#### The Wick product

In addition to a canonical vector space structure, the spaces $(\mathcal {S})$ and $(\mathcal {S})^{*}$ also have a natural multiplication given by the Wick product:

##### Definition 6.10

Let $X=\sum _{\alpha \in \mathcal {J}}a_{\alpha }H_{\alpha }$ and $Y=\sum _{\beta \in \mathcal {J}}b_{\beta }H_{\beta }$ be two elements of $(\mathcal {S})^{*}$. Then we define the Wick product of *X* and *Y* by

$$\begin{aligned} X\diamond Y=\sum _{\alpha ,\beta \in \mathcal {J}}a_{\alpha }b_{\beta } H_{\alpha +\beta }=\sum _{\gamma \in \mathcal {J}}\left( \sum _{\alpha +\beta =\gamma }a_{\alpha }b_{\beta }\right) H_{\gamma }. \end{aligned}$$

##### Example 6.11

We have

$$\begin{aligned} B(t)\diamond B(t)=B^{2}(t)-t, \end{aligned}$$

and more generally

$$\begin{aligned}&\left( \int _{\mathbb {R}}\phi (s)dB(s)\right) \diamond \left( \int _{\mathbb {R}}\psi (s)dB(s)\right) \\&\quad =\left( \int _{\mathbb {R}}\phi (s)dB(s)\right) .\left( \int _{\mathbb {R} }\psi (s)dB(s)\right) -\int _{\mathbb {R}}\phi (s)\psi (s)ds, \end{aligned}$$

for all $$\phi ,\psi \in L^{2}(\mathbb {R})$$.

**Some basic properties of the Wick product.** We list some properties of the Wick product:

1. $$X, Y\in (\mathcal {S})_{1} \Rightarrow X \diamond Y \in (\mathcal {S})_{1}$$.
2. $$X, Y\in (\mathcal {S})_{-1} \Rightarrow X \diamond Y \in (\mathcal {S} )_{-1}$$.
3. $$X, Y \in (\mathcal {S}) \Rightarrow X \diamond Y \in (\mathcal {S})$$.
4. $$X \diamond Y = Y \diamond X$$.
5. $$X \diamond (Y \diamond Z) = (X \diamond Y) \diamond Z$$.
6. $$X \diamond (Y + Z) = X \diamond Y + X \diamond Z$$.
7. $$I_{n}(f_{n})\diamond I_{m}(g_{m})=I_{n+m}(f_{n}\widehat{\otimes } g_{m}).$$

In view of the properties (1) and (4) we can define the Wick powers $X^{\diamond n}$ $(n=1,2,\ldots )$ of $X\in (\mathcal {S})_{-1}$ as

$$\begin{aligned} X^{\diamond n}:=X\diamond X\diamond \ldots \diamond X\text { (n times)}. \end{aligned}$$

We put $X^{\diamond 0}:=1$. Similarly, we define the Wick exponential $\exp ^{\diamond }X$ of $X\in (\mathcal {S})_{-1}$ by

$$\begin{aligned} \exp ^{\diamond }X:=\sum _{n=0}^{\infty }\frac{1}{n!}X^{\diamond n}\in (\mathcal {S})_{-1}. \end{aligned}$$

In view of the aforementioned properties, we have that

$$\begin{aligned} (X+Y)^{\diamond 2}=X^{\diamond 2}+2X\diamond Y+Y^{\diamond 2}, \end{aligned}$$

and also

$$\begin{aligned} \exp ^{\diamond }(X+Y)=\exp ^{\diamond }X\diamond \exp ^{\diamond }Y, \end{aligned}$$

for $$X,Y\in \mathcal {S}_{-1}.$$

Let $\mathbb {E}[X]$ denote the generalized expectation of an element $X \in (\mathcal {S})$. It coincides with the standard expectation if $X \in L^{1}(P)$. Then we see that

$$\begin{aligned} \mathbb {E}[X\diamond Y]=\mathbb {E}[X]\mathbb {E}[Y], \end{aligned}$$

for $X,Y\in (\mathcal {S})_{-1}$. Note that independence is not required for this identity to hold. By induction, it follows that

$$\begin{aligned} \mathbb {E}[\exp ^{\diamond }X]=\exp \mathbb {E}{[X]}, \end{aligned}$$

for $$X\in (\mathcal {S})_{-1}$$.

#### Wick product, white noise and Itô integration

One of the spectacular results in white noise theory is the following, which combines Wick product, white noise and Itô integration:

##### Theorem 6.12

Let $\varphi (t) \in L^{2}([0,T] \times \Omega )$ be $\mathbb {F}$-adapted. Then the integral $\int _{0}^{T} \varphi (t) \diamond \overset{\bullet }{B}(t) dt$ exists in $(\mathcal {S})^{*}$ and

6.21 $$\begin{aligned} \int _{0}^{T} \varphi (t) dB(t)= \int _{0}^{T} \varphi (t) \diamond \overset{\bullet }{B}(t) dt. \end{aligned}$$

##### Remark 6.13

Heuristically, we can see that we obtain this result by using that $\overset{\bullet }{B}(t)=\frac{d}{dt} B(t)$. If we work in $(\mathcal {S})^{*}$ this argument can be made rigorous.

### White noise for Lévy process

#### Construction of Lévy processes

The construction we did above for Brownian motion can be modified to apply to other processes. For example, we obtain a white noise theory for Lévy processes if we proceed as follows (see [7] for details):

##### Definition 6.14

Let $\nu$ be a measure on $\mathbb {R}_{0}$ such that

6.22 $$\begin{aligned} \int _{\mathbb {R}}\zeta ^{2}\nu (d\zeta )<\infty . \end{aligned}$$

Define

6.23 $$\begin{aligned} h(\varphi )=\exp \Big (\int _{\mathbb {R}}\Psi (\varphi (x))dx\Big );\quad \varphi \in ( \mathcal {S}), \end{aligned}$$

where

6.24 $$\begin{aligned} \Psi (w)=\int _{\mathbb {R}}(e^{iw\zeta }-1-iw\zeta )\nu (d\zeta );\quad w\in \mathbb {R},\quad i=\sqrt{-1}. \end{aligned}$$

Then *h* satisfies the conditions (i)–(iii) of the Minlos Theorem 6.3. Therefore there exists a probability measure *Q* on $\Omega =\mathcal {S}^{\prime }(\mathbb {R})$ such that

6.25 $$\begin{aligned} \mathbb {E}_{Q}\Big [e^{i\left\langle \omega ,\varphi \right\rangle }\Big ]:=\int _{\Omega }e^{i\langle \omega ,\varphi \rangle }dQ(\omega )=h(\varphi );\quad \varphi \in (\mathcal {S}). \end{aligned}$$

The triple $(\Omega ,\mathcal {F},Q)$ is called the (pure jump) Lévy white noise probability space.

One can now easily verify the following

- $$\mathbb {E}_{Q}[\left\langle \cdot ,\varphi \right\rangle ]=0;\quad \varphi \in (\mathcal {S})$$
- $$\mathbb {E}_{Q}[\left\langle \cdot ,\varphi \right\rangle ^{2}]=K\int _{ \mathbb {R}}\varphi ^{2}(y)dy;\quad \varphi \in (\mathcal {S})$$, where $$K=\int _{\mathbb {R}}\zeta ^{2}\nu (d\zeta ).$$

As we did for the Brownian motion, we use an approximation argument to define

6.26 $$\begin{aligned} \widetilde{\eta }(t)=\widetilde{\eta }(t,\omega )=\left\langle \omega ,\chi _{[0,t]}\right\rangle ;\quad a.a.(t,\omega )\in [0,\infty )\times \Omega . \end{aligned}$$

Then the following holds:

##### Theorem 6.15

The stochastic process $\widetilde{\eta }(t)$ has a càdlàg version. This version $\eta (t)$ is a pure jump Lévy process with Lévy measure $\nu$.

#### Chaos expansion

We assume that the Lévy measure $\nu$ satisfies the following condition:

For all $\varepsilon >0$ there exists $\lambda >0$ such that

6.27 $$\begin{aligned} \int _{{\mathbb {R}_0}\backslash (-\varepsilon ,\varepsilon )}\exp (\lambda \left| \zeta \right| )\nu (d\zeta )<\infty . \end{aligned}$$

This condition implies that the polynomials are dense in $L^{2}(\rho )$, where

6.28 $$\begin{aligned} \rho (d\zeta )=\zeta ^{2}\nu (d\zeta ). \end{aligned}$$

Now let $\left\{ l_{m}\right\} _{m\ge 0}=\left\{ 1,l_{1},l_{2},\ldots \right\}$ be the orthogonolization of $\left\{ 1,\zeta ,\zeta ^{2},\ldots \right\}$ with respect to the inner product of $L^{2}(\rho )$. Define

6.29 $$\begin{aligned} p_{j}(\zeta ):=\left\| l_{j-1}\right\| _{L^{2}(\rho )}^{-1}\zeta _{j-1}(\zeta ); \text { }j=1,2,\ldots \end{aligned}$$

and

6.30 $$\begin{aligned} m_{2}:=\left( \int _{\mathbb {R}_0}\zeta ^{2}\nu (d\zeta )\right) ^{\frac{1}{2}} =\left\| l_{0}\right\| _{L^{2}(\rho )} =\left\| 1\right\| _{L^{2}(\rho )}. \end{aligned}$$

In particular,

6.31 $$\begin{aligned} p_{1}(\zeta )=m_{2}^{-1}\zeta \text { or }\zeta =m_{2}p_{1}(\zeta ). \end{aligned}$$

Then $\left\{ p_{j}(\zeta )\right\} _{j\ge 1}$ is an *orthonormal basis* for $L^{2}(\nu )$.

Define the bijection $\kappa :\mathbb {N}\times \mathbb {N}\longrightarrow \mathbb {N}$ by

6.32 $$\begin{aligned} \kappa (i,j)=j+(i+j-2)(i+j-1)/2. \end{aligned}$$

$$\begin{aligned} \begin{array}{cccccccccc} (1) &{} &{} (2) &{} &{} (4) &{} &{} (i) &{} &{} &{} \\ \bullet &{} \longrightarrow &{} \bullet &{} &{} \bullet &{} \cdots &{} \bullet &{} \longrightarrow &{} &{} \\ (3) &{} \swarrow &{} (5) &{} \swarrow &{} &{} &{} &{} &{} &{} \\ \bullet &{} &{} \bullet &{} &{} &{} &{} &{} &{} &{} \\ (6) &{} \swarrow &{} &{} &{} &{} &{} &{} &{} &{} \\ \bullet &{} &{} &{} &{} &{} &{} &{} &{} &{} \\ \vdots &{} &{} &{} &{} &{} &{} &{} &{} &{} \\ (j) &{} &{} &{} &{} &{} &{} &{} &{} &{} \\ \bullet &{} &{} &{} &{} &{} &{} &{} &{} &{} \\ \downarrow &{} &{} &{} &{} &{} &{} &{} &{} &{} \end{array} \end{aligned}$$

Let $\left\{ e _{i}(t)\right\} _{i\ge 1}$ be the Hermite functions. Define

6.33 $$\begin{aligned} \delta _{\kappa (i,j)}(t,\zeta )=e _{i}(t)p_{j}(\zeta ). \end{aligned}$$

If $\alpha \in \mathcal {J}$ with $index(\alpha )=j$ and $\left| \alpha \right| =m$, we define $\delta ^{\otimes \alpha }$ by

6.34 $$\begin{aligned}&\delta ^{\otimes \alpha }(t_{1},\zeta _{1},\ldots ,t_{m},\zeta _{m}) \nonumber \\&\quad =\delta _{1}^{\otimes \alpha _{1}}\otimes \ldots \otimes \delta _{j}^{\otimes \alpha _{j}}(t_{1},\zeta _{1},\ldots ,t_{m},\zeta _{m}) \nonumber \\&\quad =\underset{\alpha _{1}\text { factors}}{\underbrace{\delta _{1}(t_{1},\zeta _{1})\cdot \ldots \cdot \delta _{1}(t_{\alpha _{1}},\zeta _{\alpha _{1}})} }\cdot \ldots \cdot \underset{\alpha _{j}\text { factors}}{\underbrace{\delta _{j}(t_{m-\alpha _{j}+1},\zeta _{m-\alpha _{j}+1})\cdot \ldots \cdot \delta _{j}(t_{m},\zeta _{m})}}. \end{aligned}$$

We set $\delta _{i}^{\otimes 0}=1.$ Finally we let $\delta ^{\hat{\otimes }\alpha }$ denote the *symmetrized* tensor product of the $\delta _{k}$ $^{\prime }s:$

6.35 $$\begin{aligned} \delta ^{\hat{\otimes }\alpha }(t_{1},\zeta _{1},\ldots ,t_{m},\zeta _{m}) =\delta _{1}^{\hat{\otimes }\alpha _{1}}\otimes \ldots \otimes \delta _{j}^{\hat{\otimes }\alpha _{j}}(t_{1},\zeta _{1},\ldots ,t_{m},z_{m}). \end{aligned}$$

For $$\alpha \in \mathcal {J}$$ define

6.36 $$\begin{aligned} K_{\alpha }:= I_{\left| \alpha \right| }\left( \delta ^{\hat{\otimes }\alpha }\right) . \end{aligned}$$

##### Theorem 6.16

**Chaos expansion**

Any $F\in L^{2}(P)$ has a unique expansion of the form

6.37 $$\begin{aligned} F=\sum _{\alpha \in \mathcal {J}}c_{\alpha }K_{\alpha }. \end{aligned}$$

with $$c_{\alpha }\in \mathbb {R}$$. Moreover,

6.38 $$\begin{aligned} \left\| F\right\| _{L^{2}(P)}^{2}=\sum _{\alpha \in \mathcal {J}}\alpha !c_{\alpha }^{2}. \end{aligned}$$

#### The Lévy-Hida spaces

(i) Let $(\mathcal {S})$ consist of all $\varphi =\sum _{\alpha \in \mathcal {J}}a_{\alpha }K_{\alpha }\in L^{2}(P)$ such that
  6.39 $$\begin{aligned} \left\| \varphi \right\| _{k}^{2}:=\sum _{\alpha \in \mathcal {J} }a_{\alpha }^{2}\alpha !(2\mathbb {N})^{k\alpha }<\infty \text { for all }k\in \mathbb {N}, \end{aligned}$$
  equipped with the projective topology, where
  6.40 $$\begin{aligned} (2\mathbb {N})^{k\alpha }=\prod _{j\ge 1}(2j)^{k\alpha _{j}},\text { } \end{aligned}$$
  if $$\alpha =(\alpha _{1,}\alpha _{2,},\ldots )\in \mathcal {J}$$.
(ii) Let $(\mathcal {S})^{* }$ consist of all expansions $F=\sum _{\alpha \in \mathcal { J}}b_{\alpha }K_{\alpha }$ such that
  6.41 $$\begin{aligned} \left\| F\right\| _{-q}^{2}:=\sum _{\alpha \in \mathcal {J}}b_{\alpha }^{2}\alpha !(2\mathbb {N})^{-q\alpha }<\infty \text { for some} q\in \mathbb {N}. \end{aligned}$$
  endowed with the inductive topology. The space $(\mathcal {S})^{* }$ is the dual of $(\mathcal {S}).$ If $F=\sum _{\alpha \in \mathcal {J}}b_{\alpha }K_{\alpha }\in (\mathcal {S})^{* }$ and $\varphi =\sum _{\alpha \in \mathcal {J}}a_{\alpha }K_{\alpha }\in (\mathcal {S}),$ then the action of *F* on $\varphi$ is
  6.42 $$\begin{aligned} \left\langle F,\varphi \right\rangle =\sum _{\alpha \in \mathcal {J}}a_{\alpha }b_{\alpha }\alpha !. \end{aligned}$$
(iii) If $F= \sum _{\alpha \in \mathcal {J}} a_{\alpha }K_{\alpha } \in (\mathcal {S})^{* }$, we define the generalized expectation $\mathbb {E}[F]$ of *F* by
  $$\begin{aligned} \mathbb {E}[F] = a_0. \end{aligned}$$
  Note that $\mathbb {E}[K_{\alpha }] = 0$ for all $\alpha \ne 0$. Therefore the generalized expectation coincides with the usual expectation if $F \in L^2(P)$.

We can now define the white noise $\overset{\bullet }{\eta }(t)$ of the Lévy process

$$\begin{aligned} \eta (t)=\int _{0}^{t}\int _{\mathbb {R}_0}\zeta \widetilde{N}(dt,d\zeta ). \end{aligned}$$

and the white noise $\overset{\bullet }{\widetilde{N}}(t,\zeta )$ of $\widetilde{N}(dt,d\zeta )$ as follows.

6.43 $$\begin{aligned} \overset{\bullet }{\widetilde{N}}(t,\zeta )=\frac{\widetilde{N}(dt,d\zeta )}{dt\times \nu (d\zeta )}\text { (Radon-Nikodym derivative).} \end{aligned}$$

Also note that $\overset{\bullet }{\eta }$ is related to $\overset{\bullet }{\widetilde{N}}$ by

6.44 $$\begin{aligned} \overset{\bullet }{\eta }(t)=\int _{\mathbb {R}_0}\overset{\bullet }{\widetilde{N }}(t,\zeta )\zeta \nu (d\zeta ). \end{aligned}$$

#### The Wick product

We now proceed as in the Brownian motion case and use the chaos expansion in terms of $\left\{ K_{\alpha }\right\} _{\alpha \in \mathcal {J}}$ to define the (Lévy-) Wick product.

##### Definition 6.17

Let $F=\sum _{\alpha \in \mathcal {J}}a_{\alpha }K_{\alpha }$ and $G=\sum _{\beta \in \mathcal {J}}b_{\beta }K_{\beta }$ be two elements of $(\mathcal {S})^{* }.$ Then we define the *Wick product* of *F* and *G* by

6.45 $$\begin{aligned} F\diamond G=\sum _{\alpha ,\beta \in \mathcal {J}}a_{\alpha }b_{\beta }K_{\alpha +\beta }=\sum _{\gamma \in \mathcal {J}}\left( \sum _{\alpha +\beta =\gamma }a_{\alpha }b_{\beta }\right) K_{\gamma }. \end{aligned}$$

#### The Wick product, white noise and Skorohod integral

##### Theorem 6.18

(i) Let *Y*(*t*) be Skorohod integrable with respect to $\eta .$ Then $Y(t)\diamond \overset{\bullet }{\eta }(t)$ is $dt-$integrable in the space $(\mathcal {S})^{* }$ and
  6.46 $$\begin{aligned} \int _{\mathbb {R}}Y(t)\delta \eta (t)=\int _{\mathbb {R}}Y(t)\diamond \overset{ \bullet }{\eta }(t)dt. \end{aligned}$$
(ii) Let $X(t,\zeta )$ be Skorohod-integrable with respect to $\widetilde{ N}(\cdot ,\cdot ).$ Then $X(t,\zeta )\diamond \overset{\bullet }{\widetilde{N}} (t,\zeta )$ is $\nu (d\zeta )dt-$integrable in $(\mathcal {S})^{* }$ and
  6.47 $$\begin{aligned} \int _{\mathbb {R}}\int _{\mathbb {R}_0}X(t,\zeta )\widetilde{N}(\delta t,d\zeta )=\int _{{\mathbb {R}}} \int _{\mathbb {R}_0}X(t,\zeta )\diamond \overset{\bullet }{\widetilde{N}}(t,\zeta )\nu (d\zeta )dt. \end{aligned}$$
